# "The Hospital" Nursing Mirror

**Published:** 1896-04-18

**Authors:** 


### "The Hospital" Nursing Mirror.

Being the Extra Nursing Supplement of "The Hospital" Newspaper.

[Contributions for this Supplement should be addressed to the Editor, The Hospital, 428, Strand, London, W.C., and should have the word "Nursing" plainly written in left-hand top corner of the envelope.]

### News from the Nursing World.

### ROYALTY AT BRIGHTON.

The Duke and Duchess of York during their recent visit to Brighton did not forget the hospitals. On April 10th they attended a special performance of "A White Lie," given by Mr. and Mrs. Kendal and their company for the benefit of the Sussex County Hospital. Their Royal Highnesses were received at the theatre with much enthusiasm, and after the third act Mr. and Mrs. Kendal were presented to them. Visits were also paid by the Duke and Duchess to the Metropolitan Police Seaside Convalescent Home, at Hove and to the Hove Dispensary and Cottage Hospital. Everyone will rejoice to hear that the men who were so unfortunately hurt in the accident on the previous day are reported to be going on well.

### WHOLESALE CONDEMNATION.

They are troubled by the presence of mice at the Royal Eye Hospital, Southwark, and a cat has been earnestly requested by those who are personally inconvenienced by the depredations of the little thieves. But cats are not granted admittance, even temporaril , to the hospital; there is a by-law forbidding birds, cats, &c., to be inmates of the institution. Cats have been known to carry infectious disease elsewhere, and so the whole race is tabooed at Southwark; but there is another legend which deserves some consideration, and may be regarded equally in the light of "an awful example." The scene is on the Rhine, if we remember aright, where rats infested Bishop Hatto's castle with disastrous results. Birds have not the same claim to usefulness as the domestic cat, but they are sources of genuine pleasure to patients, especially to such as are temporarily or permanently deprived of the use of their eyes. In making hard and fast rules as to pets, a committee might surely leave some loophole for an occasional exception, which might tend to the comfort of the domestic staff or the lightening of monotony for patients.

### ETIQUETTE OR CHRISTIANITY?

A storm in a tea-cup has been agitating Taunton lately over a question of clerical etiquette (save the mark)! A certain poor woman was being attended in her last illness by the nurses of the Taunton District Association, and, being much troubled in mind as her end drew near, at her desire, Miss Fisher, the lady superintendent of the association, requested the curate of another parish to visit her, to hear her confession, and give her absolution. This he did, and the woman afterwards received the Holy Communion from the curate of her own parish, who had, with the vicar, visited her during her illness. Then the fact that another clergyman had visited her became known to the vicar, who thereupon made a formal complaint to the District Nursing Committee, terming Miss Fisher's action in the matter discourteous and proselytising, and requiring an undertaking that nothing of the kind should occur again. Naturally this could not be given, seeing that Miss Fisher had broken no rule of the association, and in her private capacity was clearly acting in the spirit of the Prayer-book exhortation, which says that if any "cannot quiet his own conscience herein, but requireth further comfort or counsel, let him come to me, or to some other discreet and learned minister of God's Word, and open his grief; that by the ministry of God's Holy Word he may receive the benefit of absolution, together with ghostly counsel and advice, to the quieting of his conscience, and avoiding of all scruple and doubtfulness." That any forcing of particular doctrines by the nurses of an association is to be entirely reprobated goes without saying; but in the case at issue it was simply a matter of assisting a dying woman to avail herself of an ordinance of her own Church, and that a personal element should be brought into it is, indeed, to be lamented. It must be wondered, sometimes, where in the Gospel of Christ its teachers and preachers find a sanctionfor the things they do and say.

### NEW BUILDINGS AT LEEDS.

Improvements are on hand at the Leeds General Infirmary. A clinical theatre has lately been provided, and now the Building Committee of the Managing Board are expending much pains over the erection of two operation theatres with every possible modern adjunct. The management are also, we gladly note, contemplating the building of a proper and adequate nurses' home, the chairman having stated at the recent general meeting that it was urgently necessary for the nurses "to be accommodated under one roof and have separate bed-rooms." The staff now numbers eighty-five nurses, which may soon be increased to a hundred, and at present (we quote from the report) "they are scattered in three different places and some of them uncomfortably lodged, two or three and even four in one room." This is a terrible state of things, and ought to be remedied without the loss of a day, and as the report further states that "the considerable non-invested accumulation of some recent large legacies and handsome gifts would, it was hoped, nearly pay the cost," there seems to be no reason why this most necessary piece of work should not be taken in hand at once.

### SICK PAY FOR NURSES.

One of the members of the Royal National Pension Fund for Nurses desires to take the opportunity of her recovery from illness to express her gratitude for the handsome sick pay allowance granted her for so many weeks through the liberality of the Fund. She writes: "I have only paid six months' premiums, and the assistance I have received has been far beyond my highest expectations. I heartily wish that many more nurses could be induced to join. During the five weeks I have been in the Bognor Convalescent Home there have been no less than eight thoroughly-trained nurses, and not one of them a member of the Fund." We are requested to mention these facts, in the hope that it may induce many more than the eight nurses referred to to provide for themselves through the Royal National Pension Fund.

### IRISH WORKHOUSE REFORM.

We are informed that a conference on the above subject is to be held at St. Martin's Town Hall on Wednesday, the 29th inst., at three p.m., when the present condition of the workhouses in Ireland will be placed before the meeting by Dr. Moorhead, hon. secretary of the Irish Poor Law Medical Officers' Association. Dr. Moorhead has been working at the question for years, and it is mainly through his exertions that the matter has come under the notice of the public. Other speakers, whose names will shortly be announced, will also take part in the discussion. We hope that some permanent benefit to the unhappy paupers across the water will accrue from this conference.

### GOOD SERVICE RECOGNISED.

Miss Esther Young, who has just left Addenbrooke's Hospital, Cambridge, after holding the post of assistant matron for more than four years, will be much missed at that institution. The weekly board have placed on record their appreciation of Miss Young's valuable services, testifying that she had, during her period of office, discharged her duties to their entire satisfaction, both in her management of the probationers, and as an excellent housekeeper and organiser of the domestic work of the hospital.

### ST. PATRICK'S HOME, DUBLIN.

"Reports," remarks the compiler of the annual report of St. Patrick's Home, "are often looked upon as unnecessary and wearisome inflictions, and . . . will be consigned to the waste paper basket, unopened and unread." But no one who looks beyond the first page is likely so to treat the publication in question, for it is full of interest. St. Patrick's Home for Supplying Trained Nurses to the Sick Poor is affiliated with the Q.V.J.I.N., and warm praise and appreciation of the work accomplished under its auspices for district nursing in Ireland has been expressed by Miss Dunn, general superintendent in Ireland of Queen's nurses, and by Miss Peter, who paid two visits to the home in 1895. The staff now numbers thirteen, including the superintendent and probationers, and no less than 25,284 visits were paid during the past year. A good idea of what is comprised in an ordinary working day may be gathered from the diary of one of the nurses, quoted in the report, and this shows, too, the value of two associations in connection with the home, the Samaritan Fund and the Needlework Guild, which supply extra comforts for the very poorest cases, and help to provide bandages, splints, and crutches, and many modern appliances for the relief of suffering. With regard to expenditure, the Jubilee grant is only sufficient to defray the cost of the Queen's probationers, four of whom are trained every year, and the home therefore relies entirely for support on the contributions of subscribers. A calculation was made lately as to the actual cost of each visit paid, which worked out at the surprisingly small sum of tenpence. The account of all that is being accomplished by St. Patrick's Home (which was, by the way, the second home for trained district nurses established in the United Kingdom) ought to encourage old friends to liberal help, and gain for it new and warm supporters. Much of the successful record is doubtless due to the wise and kindly rule of Miss Franceys Howell, the valued superintendent.

### NURSING IN THE UNITED STATES.

A correspondent informs us that New York, like most other large American cities, is suffering from a plethora of trained nurses. She declares it to be impossible to say what will be the outcome of the present tendency to increase the nurse training school accommodation everywhere, and to turn out increasing numbers of trained nurses every year, who cannot hope to make a living by the exercise of their calling in the United States. The three large training schools in New York, *i.e*, the New York Hospital, the Presbyterian, and St. Luke's, have done something by adopting the three years' course, and we should be glad to have further and fuller information on this interesting aspect of the future of nursing in the United States.

### STOCKPORT UNION INFIRMARY.

The Board of Guardians have purchased a large house adjacent to the boundary wall of the workhouse, which is to be converted into a nurses' home. This will no doubt remove the long-felt grievance of want of accommodation for nurses. It is to be hoped that in rearranging and fitting up the house the Guardians will obtain the advice of a practical man and an experienced matron.

### SHORT ITEMS.

In response to a request from King Menelik of Abyssinia, that his country may be admitted to the Red Cross Convention, it is said that the Russian Red Cross Society has sent to Abyssinia a number of surgeons and nurses with medical supplies.—The District Nurse Committee have arranged for lectures on "Good Nursing and How to Get it," at Towcester at 3 p.m. snd 7.30 p.m. on Thursday, April 23rd. The lectures will be given by Miss Gethen, formerly Sister Queen, London Hospital.—The Bideford Board of Guardians have raised the salary of Nurse Arnold from £25 to £30. Universal praise was accorded to the manner in which Nurse Arnold carried out her work amongst the aged and infirm folk in the infirmary, by whom she is regarded with sincere affection. — The Society for Promoting Christian Knowledge have brought out a small pamphlet by Mrs. Ernest Hart, entitled "Vaccination and what it does, simply explained." Useful and interesting information will be found in its pages.—Three ladies belonging to Lady Roberts' Nursing Staff left England last month in the ss. *Rufford Hall*, to nurse in the military and officers' hospitals in the Punjab, India; Miss Ellen Harris, formerly a lady superintendent in the Indian Nursing Service, has also lately joined Lady Roberts' staff of nurses.—Miss M. Christie, M.B.Lond., has been elected junior medical officer of the Greenwich Workhouse, at a salary of £80 per annum. There were three women among the twenty-three applicants for the post.

### Hygiene: For Nurses.

By John Glaister, M.D., F.F.P.S.G., D.P.H.Camb., Professor of Forensic Medicine and Public Health, St. Mungo's College, Glasgow, &c.

### II.—THE HOUSE WE LIVE IN.

### The Sick Room.

In the preceding article the subject of "elbow-room" was partly discussed. A few words still remain to be said, however, on this subject. If two rooms contained 3,000 cubic feet, and were occupied by a family composed of father, mother, and six children, the amount of cubic space available for each—even counting two of the youngest children as one adult—would be less than a room of 1,000 cubic feet occupied by two adults. The amount of ventilation necessary to supply the two apartments with an adequate supply of fresh air must necessarily be much greater than that for the one apartment. This will be clearer when the subject of ventilation comes to be discussed, as will also the point whether it would be possible to safely ventilate these apartments in consonance with the health requirements of the inmates. Hence the relation of the amount of cubic space per individual to the possibility of ventilation becomes a question of great importance. It may be taken as an axiom that the smaller the apartment the greater is the difficulty of sufficient and safe ventilation; in like manner the smaller the amount of cubic space allotted per individual the more difficulty is experienced in solving the problem of supplying the necessary amount of air to maintain health. The cubic space of a room in reference to ventilation has always an important bearing on the health of the occupants at any period of the time of occupancy, and this bearing is a thoroughly practical one. It is quite true that in considering the theoretic amount of air required per person, the question of cubic space loses some of its importance at the end of the first hour of occupancy, but it is always of importance in reference to the practicability of supplying the necessary amount of air.

When illness of any kind enters a house, if the patient must needs be confined to bed, it becomes of prime importance that the room best suited for his treatment should be devoted to that end. Where there is comparatively little spare space in the house, it is often advisable to convert one of the sitting rooms into a bedroom, particularly if it be more suitable in certain respects than the other rooms. If, however, the house be of more than one flat, usually a bedroom on the upper storey is preferable to one on the lower. The patient is less likely to be disturbed by household operations, the sick-room slops can be more easily disposed of in the adjacent bath-room, and the atmospheric conditions of the sick-room are liable to less disturbance, being further away from the doors opening to the outer air. The conditions which should be looked for in the required room are these, viz., it ought to be well lighted by a window which is capable of being opened under certain precautions to admit of the entrance of fresh air from time to time; it should contain a good fireplace, which will exhaust the lower strata of air of the room when the fire is in operation; and it ought to be convenient to the bathroom, for disposal of sick-room slops. Should there be anything special in the nature of the ailment calling for special treatment the room should be chosen accordingly. If, for instance, the patient be a nervous one, or suffering from insomnia, the situation of the room must be such as will procure the greatest amount of quiet. Here the question of light becomes of less importance, although that of ventilation remains. These general conditions will suffice for the ordinary run of illnesses. When, however, infectious disease makes its appearance in a house, not only is the same attention to be paid to the patient's comfort, but a new element enters into the problem, viz., the comfort and safety of the other occupants of the house. Equally of importance with the care of the patient is the prevention of the extension of the disease to others, both inside and outside the house. Under these circumstances, the position of the room occupied by such a patient becomes of the chiefest importance. It must be removed as far as possible from the living rooms of the other occupants, and it must also be in proximity to the bathroom, so that sick-room utensils may be amply disinfected, sick-room clothing be steeped in germicide liquid, the patient be adequately supplied with water for cleansing purposes, and the nurse for disinfecting herself preparatory to taking exercise out of doors. Contagium is a particulate substance, and cannot pass through doors or walls. That is true. Wherefore, then, the necessity for all these precautions? Because this particulate matter is so light that it is easily air-borne, and may be thrown into the common air supply of the other rooms on opening and shutting the sick-room door; besides, it adheres to everything that proceeds from the sick-room. Hence the above necessary precautions.

The duties of the nurse in the sick room, in addition to those necessary for the personal comfort of the patient and to those laid down by the physician, include the proper ventilation of the room, and the maintenance of a suitable temperature, during her term of duty. The ventilation of the ordinary sick-room is by no means a simple matter; indeed, it is rather a difficult duty to perform. "Not at all," says some one; "it only means the opening of the window." If that were all, too much fuss has been made regarding ventilation. Opening of windows without special precautions, spells draughts; draughts mean danger to patient, if not, indeed, actual mischief. Suppose that the temperature of the room has been maintained at 65 degs. Fahr., and that the temperature of the outside air be about 30 degs. Fahr., the air—being so many degrees colder—will rush in by the opened window as does a torrent of cold water, with a quickened velocity, because of the difference of the two temperatures. This will become more apparent to the reader later. There are, however, ways by which safe ventilation may be obtained by means of the window, which will be discussed in due course.

So likewise is the maintenance of a proper temperature in a sick room of great importance. A routine temperature, say 60—65 degs. Fahr., is not suitable for all classes of diseases. A room temperature suitable for a convalescent who cannot part safely with much body heat would not be grateful to one suffering from high fever. In like manner a comparatively dry atmosphere would be less suitable to a "croupy" patient than a humid one.

These general considerations are set down to indicate how much less simple than is usually thought such points are.

### The Nursery.

There was a time when any odd room in the house was thought good enough for the daily life of the little ones. Such a time is now, fortunately for the children, nearly over. The nursery ought, indeed, to be one of the best, one of the largest, one of the brightest rooms in the whole house, situated, if you like, and perhaps preferably, in the top of the house, because very often there the light is best and most sunlight is to be obtained. After appropriate feeding, probably the next most important thing which is conducive to healthy child life is abundance of light and sunlight. Like the plant deprived of sunlight which does not develop the chlorophyll—the green colouring matter—in its leaves, so the child grows up pale and sickly-looking; and not only sickly-looking, but actually in depreciated health.

The Italian proverb, "Where the sunlight does not go, the doctor does," is very true; and the light seems as necessary for the proper development of the red colouring matter of the blood of the child as for the green colouring matter of the leaf of the plant.

In addition to the best light, let there be free elbow-room. The animal life of the young is considerable. Romping is as natural and as necessary as eating and drinking, and, while confinement is irksome to all, it is much more so to the child.

Of equal importance to the two foregoing is the temperature of the nursery. Children are like sensitive thermometers; their bodies register a falling temperature nearly as quickly as do these instruments. Parting with their body heat rapidly, they are extremely susceptible to outside frigifactive influences. Much harm has been produced from ignorance on this point, and many children have received fatal illnesses from mistaken notions on the part of parents and nurses in regard to the means necessary to make them hardy.

Well lighted, large spaced, appropriately heated, let the nursery be, and the children will thrive into vigorous health.

### Royal British Nurses' Association.

### QUARTERLY MEETING.

The quarterly meeting of the general council of the Royal British Nurses' Association was held at the offices of the corporation, Old Cavendish Street, on Friday, April 10th, at five o'clock. The chair was taken by Sir James Crichton Browne. Mr. Pickering Pick, Dr. Bezley Thorne, Mr. Pearce Gould, Miss Wedgwood, Miss Cartwright, and some 30 or 40 members were present. Miss Ravenhill was absent through ill-health.

The financial report from January to March, 1896, presented by the hon. treasurer, Mr. Langton, stated that during that quarter the sum of £302 11s. 4d. had been received, and £347 7s. 10d. disbursed, and that "a not inconsiderable portion of the expenditure had been due to the payment of some charges in connexion with the lawsuit of Barlow v. The Corporation, which accounted for the sum of £128 3s. 7d." There was an amount due on account of this action of £125 still remaining to be paid.

Dr. Bedford Fenwick objected to the words "Barlow v. The Corporation," which he considered an inaccuracy, and desired the substitution of "Barlow v. Thorne and others," which verbal alteration was subsequently agreed to; and the business of the meeting was further delayed for some minutes while the reasons for the accounts being audited to March 25th instead of March 30th were explained again.

The report of the Executive Committee was then read by Mr. Fardon, summarising the business of the past quarter. Mrs. O'Callaghan asked when Sir J. Russell Reynolds was elected a member of the Association. He was not a member at the time of the special meeting convened in January to consider the Barlow case, and was not therefore in order in moving a resolution. Sir James Crichton Browne replied that he was an ex-officio member in the capacity of President of the Royal College of Physicians, and the executive had the opinion of two eminent counsel to the effect that his action was perfectly in order.

A series of trivial objections were then raised by Dr. Bedford Fenwick, which it is unnecessary to comment upon at length, almost every clause in the report coming under the ban of his displeasure in turn. He referred to Miss Wedgwood's action in voting at the Conference held at the instance of the British Medical Association in January to consider State registration, protesting that by her voting against State registration she was acting against the avowed principles of the Association, to uphold which that Association took money from its members. A new regulation as to training:—"That all nurses who commence their training on and after October 1st, 1896, will be required to conform to the resolution of the Council that the training must be in hospitals containing not less than 40 beds"—aroused Dr. Bedford Fenwick's deepest indignation. It would, in his opinion, make the Association ridiculous in the eyes of the world, and what would be said by the managers of hospitals of less than 40 beds, he really could not think. The proposal to hold the next annual meeting in London instead of in the provinces next came in for blame. It was, said Dr. Bedford Fenwick, a distinct promise made to the members of the Association that the meetings should be held in the provinces. To this remark, however, there was general dissent on the part of the meeting.

Ultimately, after explanations and reasons had been given by Mr. Fardon which fully commended themselves to the general sense of the meeting, and after some discussion and much waste of time, the report was adopted almost unanimously, Dr. Bedford Fenwick finding only three nurses and Mrs. O'Callaghan to support his counter resolutions.

The re-election of the council was the next business, and printed nomination lists were handed round. This list again gave rise to more criticism from Dr. Bedford Fenwick; especially he took exception to the number of nurse members proposed from Middlesex Hospital and Chelsea Infirmary; their preponderance, he considered, meaning nothing less than the "formation of a ring."

By this time Dr. Bedford Fenwick's interruptions and objections had exhausted the patience of the meeting, and more than one protest was made against the waste of time occasioned thereby. General relief was therefore felt when Sir James Crichton Browne moved that the nomination list be accepted, which was promptly settled by an overpowering show of hands, and finally remarked that Dr. Bedford Fenwick was really taking a little too much upon himself in undertaking, as he appeared to do, to speak for the whole of the medical profession. At that meeting, at all events, he stood absolutely alone. With which incontrovertible statement of fact the meeting broke up.

It would appear that with regard to Dr. Bedford Fenwick's objection to the nomination list, all the matrons on the council were invited to nominate members, but only two, Miss Thorold and Miss Cartwright, did so.

### A REGISTERED UNIFORM.

The Secretary of the Royal British Nurses' Association requests us to state that a book is now lying at the offices of the association, 17, Old Cavendish-street, London, W., to receive the signatures of those in favour of, or opposed to, the adoption of a registered uniform for the optional use of members of the Royal British Nurses' Association.

### Where to Go.

Swiss National Exhibition at Geneva.—This exhibition is to be held in the summer, and the structural arrangements are approaching completion. It will prove an additional attraction to tourists to Switzerland, and very cheap railway arrangements will be made in connection with it.

Society for Waifs and Strays.—A drawing-room sale of work in aid of the above society is to be held at Lady Wellesley's, 17, Chester Square, on Friday, April 24th, from three to seven p.m. Admittance free. Tea and coffee.

### Wants and Workers.

[The attention of correspondents is directed to the fact that "Helps in Sickness and to Health" (Scientific Press, 428, Strand) will enable them promptly to find the most suitable accommodation for difficult or special cases.]

A correspondent wishes to know of a "cheap, simple book dealing with English plants used for medicinal purposes." Can any reader supply information?

### Trained Nurses' Clinic.

### II.—WHAT TRAINING MEANS.

### The Present Day.

Although certain qualifications are demanded in the would-be probationer, no special provision has been made for her preliminary education except in a few places, notably Glasgow Royal Infirmary and the London Hospital, Whitechapel.

At Glasgow certificates for clinical work must be obtained by candidates before they enter the wards of the Royal Infirmary as probationer-nurses. These certificates are granted after an examination in anatomy, physiology, and hygiene, which are the subjects comprised in the first course of education in nursing. The scheme is said to have worked well, and the period occupied by this first course forms a better test of the candidates' capacity than the more general plan of a month's trial in the wards.

In connection with the London Hospital Nursing School, preliminary training for probationers is provided at a house specially adapted for the purpose, and, in addition to the three subjects taught at Glasgow, instruction is given in such portions of ward work as will be a part of their daily routine in hospital. They are expected to become proficient in bandaging, sick-room cookery, and all such details of practical nursing as can be taught by way of preparation for their actual attendance on the sick. At the end of six weeks examinations are held in the various subjects, and if these are passed satisfactorily the pupils can enter on a three years' engagement in the hospital. No certificates are given after the preliminary examination, but a record is kept and the result embodied in the certificate granted on the completion of the whole training. Those who have passed through this course have already proved its practical benefit in equipping them to enter on the regular hospital training with comfort to themselves, their fellow workers, and the patients.

To those interested in the wonderful advancement made in trained nursing during the last dozen years the prospectuses of many schools of nursing afford gratification. At King's College Hospital there are two distinct courses of lectures, the first being elementary and the second for the probationers in their second year. One of the most elaborate and thorough systems for the education of nurses is in force at the Prince Alfred Hospital, Sydney, N.S.W., where examinations are held at the completion of each year during the course of training. The range of subjects is most exhaustive, and includes nearly every branch of nursing, and many studies followed in England only by students of medicine.

But the true training of a nurse also embraces many important things which are liable to be overlooked amidst the elaborate details of her present education. To the intelligent woman who takes to nursing at or after the age of twenty-four, it should be obvious that she must not rest content with what is casually taught to her. She must learn many other things, and she must train her own mind and body. Although she must concentrate her attention on her work, never allowing anything to come between her and her patients whilst she is on duty, yet she should have interests apart from those of the ward. When she leaves her patients she should not brood on them, but should rather store her mind with other matters, fitting herself to return to work mentally refreshed by new ideas and physically strengthened by fresh air and outdoor exercise.

Beyond all other things a nurse needs to be sympathetic, and it is very doubtful whether anyone can be a satisfactory nurse without this quality. That alone can make her acceptable to the sick, and enable her to serve them faithfully.

Neither technical nor theoretical knowledge can take the place of love of humanity and pity for all sufferers. Personal influence is nowhere more felt than in the sick room, and every woman who adopts the calling of a nurse needs to keep her responsibilities in view, as well as her actual proficiency in her art. Neither patients nor nurses are mere machines, hence it is undesirable that the individuality of either should be ignored.

In striving for knowledge of all kinds, bearing directly or indirectly on her work, it is of the first importance that true womanliness should be preserved, as the sympathetic voice and touch do more to reconcile the sick to their helplessness than the somewhat pretentious qualifications which are considered essential for the modern trained nurse.

### Our American Letter.

April will see the opening of several new buildings in the United States. These include the erection of a new wing to the Sisters' Hospital, Main Street, Buffalo, which will consist principally of private rooms, with ward accommodation for eighteen beds. Promises have been received from many friends to furnish these wards and rooms, and the authorities hope it will be ready for patients very soon after Easter. A new hospital has been built as an adjunct to the New York Homœopathic Medical College and Hospital, and is very nearly ready for occupation. The building has cost 95,000 dols. There are male and female wards (of which the fittings have been supplied by Mrs. C. T. Kunhardt and Mrs. E. C. Benedict), with a maternity department on the third floor, and a certain number of private wards. The fourth floor will be devoted to the accommodation of the nurses and servants. A new maternity ward is being added to the City Hospital, Lynn, Mass. A house has been taken for this purpose very near the main building, and will be fitted up as promptly as possible. The Stamford Hospital, too, for which 80,000 dols. was subscribed, and a sum of 2,500 dols. received as a grant from the State, will be opened in a few weeks. Plans have also been completed for an addition to the General Hospital, New Haven, Conn. The new building is intended to serve mainly as a nurses' home; it will also contain a kitchen on the first floor, specially fitted with every appliance for preparing invalid food. This annexe will be of red brick, in architectural conformity with the rest of the hospital, and it is anticipated will cost between 6,000 and 7,000 dols. The directors of the U.P. Women's Association, Pittsburg, Pa., have decided to build a new memorial hospital for children in Alleghany, to cost between 35,000 and 60,000 dols. The question of a site is now under consideration.

Efforts are being made by the Women's Society for the Promotion of the Welfare of the Insane to obtain the admission of women members to the State Commission in Lunacy and to the boards of management of State hospitals, and for permission to have medical women appointed to serve in the hospitals. A statement has been made before the Assembly General Laws Committee on the subject by Dr. Phœbe J. B. Waite and Dr. Amelia Wright, of New York City. This commission is now considering the revision of the Lunacy Laws of the State.

Miss Edith Compton has been compelled to resign her work as superintendent of the Protestant Infirmary, Lexington, Kentucky, on account of ill-health. Her place has been filled by Miss Emma E. Mathias, late assistant matron at the Grady Hospital, Atlanta, Georgia. Both these ladies are graduates of the Episcopal Hospital Training School of Philadelphia. Much regret has been caused among her fellow-workers by the resignation of Miss Emily Lawson Jones of the position of head nurse at St. Elizabeth's Home for Incurables and Convalescents, Providence.

The Alumnæ Association of the New York Training School for Nurses at their last monthly meeting discussed the subject of "Provision for Sick Members." Mrs. Ollesheimer, vice-president of the Working Girls' Mutual Benefit Fund, a society which has existed for five years in connection with the working girls' clubs in New York, gave an address enumerating the benefits to be derived from such an association. An associate membership has lately been organised which would embrace nurses in its provisions. A Bill is now before the Legislature with the object of providing new buildings, in which shall be special accommodation for the sick nurses of the training school.

A training school in connection with the State Hospital for the Insane, at Morganton, North Carolina, has been recently established, of which Miss S. E. Pitts, a graduate of the South Carolina Asylum Training School and of the Massachusetts General Hospital, has been placed in charge. A two years' course is offered to pupils, which will combine with general hospital training special instruction in the care of the nervous and insane. Eventually it is proposed to extend the instruction to the male nurses in the hospital. In this connection, by the way, it is not perhaps generally known that there is a male training school attached to the St. Peter State Hospital, Minn. It is frequently stated that there are two such schools in the United States, but this third one is overlooked.

### The Nurse Gossip.

The following letter was published in the *Times* of April 13th, and we reproduce it in our columns because although the accusation of sick-room gossip, now, alas! often too true, is certainly not an attribute of the modern nurse especially, we feel that the stigma is one which should be removed from a noble calling. It is well to see ourselves as others see us, sometimes, and the exercise of a little self-discipline and thought will prevent much permanent harm:—

"Sir,—As your correspondents on the above subject have been so far presumably men, will you allow me as a woman to inquire what is to become of women generally if we are to begin troubling about privilege, defamation, and such troublesome subjects? Is not the tedium of the sick-room immensely relieved by the prattle of the sick nurse, and monthly—lovable beings who are quite as deeply initiated into the secrets of the sick-room as the doctor, and without any of the disagreeable fears that generally haunt him on the score of honour and consequences? So long as we can get all the information we can possibly desire through this fertile source, and a thousand other little channels known to every female inhabitant of the globe, we can afford to see the ladies' doctor locked up under the seal of confession without finding ourselves any the worse. In rural districts the trained nurse is considered a perfect godsend to the place until some busybody in high life awakens to the fact that she is the bee that carries the pollen of unrighteous gossip from door to door, and dismissal follows at the interesting moment when the dull village is converted into a lively hotbed of scandal. To sum up, you and the law may do as you please with the doctors, but spare, oh, spare us, the modern trained nurse."

### Generous Help.

The directors of the Paisley Infirmary are to be congratulated. It is stated that Mr. Peter Coats, of Ferguslie Thread Works, has promised a donation of £10,000 towards the £63,000 for which an appeal has been issued with the object of erecting a new hospital. Mr. Coats had previously subscribed £5,000, and is also building for the infirmary a new nurses' home at a cost of about £10,000. With the way shown in this generous manner the rest of the money needed ought to be speedily collected.

### Appointments.

Walton-on-Thames Convalescent Home.—Miss Edith Sutcliffe has been appointed Matron of this institution. She was trained at St. Mary's Hospital, Paddington, and has since held the post of matron at the Hospital for Women and Children, Leeds, and the Royal Bath Hospital and Rawson Convalescent Home, Harrogate. Miss Sutcliffe has our best wishes for success in her new work.

Macclesfield General Infirmary.—Miss Mary E. Noble has been appointed Matron of this infirmary. She was trained at the Children's Hospital, Nottingham, and the Royal Infirmary, Bristol. She has since held the post of sister at the Children's Hospital, Bradford, for three years, and worked at Northampton General Infirmary as sister of the male wards. During the last three years Miss Noble has been sister-in-charge of the Chorley Dispensary and Cottage Hospital. We congratulate Miss Noble on her appointment, and wish her all success in her new work.

### Notes and Queries.

The contents of the Editor's Letter-box have now reached such unwieldy proportions that it has become necessary to establish a hard and fast rule regarding Answers to Correspondents. In future, all questions requiring replies will continue to be answered in this column without any fee. If an answer is required by letter, a fee of half-a-crown must be enclosed with the note containing the enquiry. We are always pleased to help our numerous correspondents to the fullest extent, and we can trust them to sympathise in the overwhelming amount of writing which makes the new rules a necessity. Every communication must be accompanied by the writer's name and address, otherwise it will receive no attention.

### Queries.

(12) *Assistant Nurse.*—Will you tell me what is meant by the term "assistant nurse," and what position she takes?—*Nurse G.*

(13) *Fever Nursing.*—Will you tell me if I can obtain training at any of the M.A.B. hospitals for a period of from six to nine months? I hold a two years' first-class certificate from Guy's Hospital for general nursing.—*Muriel.*

(14) *Fever Nursing.*—Are there any fever hospitals in or near London where I could be trained as a probationer? I am 24 years of age.—*E. M. C.*

(15) *Nurse Dolls.*—Where can I get any information about the nurse dolls?—*Derbyshire District Nurse.*

(16) *Training.*—Will you please tell me where I can get an introduction into a general hospital, London or country, to be trained as a probationer?—*L. E. P.*

(17) "*How to Get into a Fever Hospital.*"—Will you please tell me in which number of The Hospital an annotation with this title is to be found?—*M. F. H.*

(18) *Uniform.*—Can you tell me where to write to obtain patterns for making nurse's outfit?—*A. P.*

(19) *A Puzzle.*—Please say in your next issue if it requires an educated person to go as a probationer?—*B. M. B.*

### Answers.

(12) *Assistant Nurse* (*Nurse G.*)—The term "assistant nurse" means, we fancy, different things in different institutions. It is usually applied to women who enter an institution in virtue of some previous training not quite as probationers, and yet distinct from the staff or charge nurses, who are fully qualified and certificated. The larger hospitals do not recognise the term in most cases, holding that whatever training may have been received previously, in their wards a new comer is only a probationer until she has gained the certificate, granted by the Training School to those only who have gone through a certain course extending over two or three years, as the case may be.

(13) *Fever Nursing* (*Muriel*).—You will find a list of the M.A.B. Fever Hospitals in Burdett's "Hospitals and Charities," with the names of the matrons. Write direct to them.

(14) *Fever Nursing* (*E. M. C.*)—See reply under the same heading above.

(15) *Nurse Dolls* (*Derbyshire District Nurse*).—We have received many inquiries about the Nurse Dolls of late, and are sorry to be obliged to say that they are not just now at liberty to pay visits.

(16) *Training* (*L. E. P.*)—Read the note at the head of this column with regard to answering letters by post. Probationers do not obtain admission into hospitals by "introductions," but by giving evidence of suitability for the work they wish to take up. Read "How to Become a Nurse" (Scientific Press, 428, Strand, W.C.), and if your age is within the prescribed limits, write yourself to the matrons of the various hospitals, of which you will find a full list given.

(17) "*How to Get into a Fever Hospital*" (*M. F. H.*).—The Hospital for December 14th, 1895, p. 179.

(18) *Uniform* (*A. P.*).—Debenham and Freebody, Wigmore Street, Cavendish Square, W., or Garrould, Edgware Road, are well known firms which make a speciality of nurses' uniforms, and either will probably be able to supply you with whatever "patterns" you need.

(19) *A Puzzle* (*B. M. B.*).—Certainly "it requires an educated person" to become a nurse, that is to say, a fair general education and intelligence are indispensable. "A knowledge of Latin and Greek" is not the rule, as one correspondent seemed to think a little while ago; but there are examinations written and oral to be passed in the elements of anatomy, physiology, and hygiene which belong as much to the training of a modern nurse as the more strictly practical work of learning to make beds and poultices.

### A Book and its Story.

"A LADY OF QUALITY."*

The period of this novel is the end of the eighteenth century. Sir Jeoffrey Wildairs, of Wildairs Hall, "a large man, of florid good looks, black eyes, and full habit of body,' is a type of the deep-drinking English squire of those days. He has a grudge against his wife because no son and heir has been born to them; and when the story opens, on a windy morning at the close of the year 1690, he hears, as he is setting out to hunt with a party of his boon companions, that another daughter has just been added to his family. He refuses to go and see his wife. "Bedamned to her!" he exclaims, and gallops off. In a huge chamber, hung with tattered tapestries, my lady lay in a gloomy, canopied bed, with her new-born child at her side. The hired nurse has deserted her, the mother lies alone; and "when the nurse came back, the chamber was cold as the grave's self—there were only dead embers on the hearth, the new-born child's cries filled all the desolate air, and my lady was lying stone-dead, her poor hand resting on her offspring's feet, the white of her open glazed eyes seemed to stare as if in asking Fate some awful question."

The child thus born was named Clorinda, and is afterwards the Lady of Quality whose story is told in these pages. In her early years she was ignored by her father, and lived, with her two sisters, in the barren, ill-kept rooms of a remote wing of the home. Sir Jeoffrey was such a careless father that he did not even know her by sight. She spent her time in the company of grooms and maidservants, "she played in the kennels and among the horses' heads, and learned to use oaths as roundly as any Giles or Tom. There was no human creature near her who had mind or heart enough to see the awfulness of her condition, or to strive to teach her to check her passions; and in the midst of these perilous surroundings the little virago grew handsomer and of finer carriage every hour."

At last, one day her father found her playing with a flask of gunpowder, and boxed her ears. In an instant she seized a whip, flew upon him, swearing at him and hitting him with all her childish strength. He burst out laughing, and, as the servants ran in, exclaimed, "Who is the young she-devil? Ods bodikins, who is she?" Being told that she was his youngest daughter, he caught her up in his arms, "A fiercer little devil for thy size I never saw—nor a handsomer one," he said, and from that moment the child and he were inseparable. He had her dressed like a boy, took her everywhere with him, and showed her off to his friends. This went on until her fifteenth birthday, when Sir Jeoffrey gave a great dinner. "When the men trooped into the black oak wainscotted dining-hall on the eventful night they found their audacious young hostess awaiting them in greater and more daring beauty than they had ever before beheld. She wore knee-breeches of white satin, a pink satin coat embroidered with silver roses, and white silk stockings. She met them standing jauntily astride upon the hearth, her back to the fire, and she greeted each one as he came in with some pretty impudence. Her black eyes were like loadstars, drawing all men, and her colour was that of a ripe pomegranate."

Clorinda was used to the company of rollicking squires, and could give them back their coarse jokes with interest, but on this occasion there was a young gallant with hands as white as her own, with a fashionable coat, and fair, flowing locks, which scattered some delicate French perfume. This was Sir John Oxon, a professed lady-killer. He was much attracted by her. At this dinner she drank toasts and played the boy for the last time; and after that she always dressed as a woman, and took her place in society, when she had admirers in plenty.

We get some slight hints of a *tendresse* for Oxon, and when she heard that he was engaged to marry a heiress there is a scene, in which she flouts him, and they part, not on the best of terms. At last she accepts the addresses of Lord Dunstanwolde, a man of mature years. It is at a ball in London that she promises to marry him; and as she walks along leaning on his arm, with all eyes upon her, and her beauty shining at its highest, her future husband presents to her a gentleman—"My honoured kinsman," he says, "His Grace the Duke of Osmonde."

She had given her promise to Dunstanwolde, but from the moment Osmonde's eyes met hers, she had known that 'twas the only man's eye before which her own would fall, and which held the power to rule her very soul. "Here was he who, had she met him one short year before, would have revolutionised her world."

Nevertheless she is a very true and faithful wife to Dunstanwolde; and Osmonde, though he is passionately devoted to her, keeps aloof, so that nothing mars the domestic peace of the establishment in which she maintains her character of the Lady of Quality until her husband's sudden death. Then Osmonde presents himself as a suitor, and later a marriage is arranged between them. But Sir John Oxon, whose marriage has been broken off, also pays his addresses to her. She keeps him at a distance; till one day he obtains a private interview with her, the nature of which may be gathered from what passes between them. She proclaims her love for Osmonde. "There is no other man on earth," she cries; "were he a beggar, I would tramp the high road by his side, and go hungered with him. He is my lord, and I his mate—his mate!"

"That you will not be," he answered, made devilish by her words. "He is a high and noble gentleman, and wants no man's cast-off plaything for his wife."

Her breast leaped up and down in her panting as she pressed her hand upon it; her breath came in sharp puffs through her nostrils.

"And once," she breathed—"and once—I loved thee—cur!"

He was mad with exultant villainy and passion, and he broke into a wild laugh of derision. "Loved me!" he said, "thou! as thou loved him—so will Moll Easy love any man—for a coronet."

This is the crisis of the story; when Oxon threatens to expose the Lady of Quality to the man she loves; but to discover the means by which she extricates herself from the web in which her past life has involved her, and the way in which the plot is wound up, must be left to the reader.

As a picture of manners in the reign of Queen Anne the book is, on the whole, accurate in detail, and the novel is not only a romance of a high order, but is undoubtedly the best piece of work which Mrs. Burnet has produced. Though the Lady of Quality is the central figure, some of the minor characters are finely drawn, particularly that of Anne, who, knowing the secret of her sister's life, conceals it with loving fidelity.

* "A Lady of Quality." By Frances Hodgson Burnet. (London: Frederick Warne and Co. 1896.)

### Presentation.

At the Greenwich Infirmary, on April 8th, John Howell Griffith, M.B., B.S., was presented by Dr. Burney (medical superintendent), on behalf of the staff, with a handsome dressing-case, fitted with ebony and silver. During Dr. Griffiths' term of office (over two years) he has been much liked, his clinical lectures to the nurses being greatly appreciated. His loss will be sincerely felt.

### Everybody's Opinion.

[Correspondence on all subjects is invited, but we cannot in any way be responsible for the opinions expressed by our correspondents. No communications can be entertained if the name and address of the correspondent is not given, or unless one side of the paper only be written on.]

### HOLIDAY HOMES FOR NURSES.

"A. E." writes: In your two last numbers of The Hospital I see a query about a "holiday home for nurses" in Hastings. Does "Nurse R." make a mistake and mean Brighton? There is a charming home there for nurses taking a holiday called the Home of Rest for Nurses, 17, Sussex Square, Brighton. Spending a day or two in Brighton this month, I visited this home. It is in a very good situation, luxuriously furnished, has a good library, and the lady in charge seemed to me quite charming—she was so kind showing me everything. Not having seen this home mentioned in The Hospital, I thought you might like to know about it. The terms, as far as I remember, are 15s. a week single room, 21s. double-bedded room.

[We believe that admission to the Home of Rest at Brighton is limited to members of the Royal British Nurses' Association.—Ed. *T. H.*]

### WANTED, GOOD FEVER NURSES.

"A Looker-on" writes: There is an old trite aphorism which tells how the looker-on always sees the best part of the game. Age has robbed it of none of its force, neither has time diminished its truth. May I, then, as a looker-on, be permitted, without presuming, to point out at least one, if not more, reasons why the above heading appears so frequently in the advertising columns of the nursing press? The view you took in a recent number, that it is not due to the fear of infection, is not likely to be disputed, or that either the pay, holidays, food, and quarters as a rule are good. There is, however, another reason, farther reaching and much more important—and that is the narrow limit placed upon women of ambition. How rarely, if ever, do we find the higher posts being given to those who have devoted their best days of work to the study of infectious illness? Usually Sisters and matrons are elected from a class who count upon a sound general training as the first and almost only consideration—fever a very minor one. Once let this tradition be observed more in the breach than its performance; once let it be known that, all things being equal, vacancies in the higher ranks shall be filled by specialists in that branch of the service, and then the heading of this letter will only be conspicuous by its absence. An inducement would be offered to the best of the profession to enter for fever work, knowing that in time their right would be recognised to share in the good things of the profession. Until this is done there will always be a dearth of nurses, for none really desire to enter a field where their scope is so limited.

### NURSING POLITICS.

We have received the following letter from Nurse Jane Snowden, a member of the Registered Nurses' Society: Will you kindly allow me space in your paper for a few words in reference to an article in your columns this week, headed "Nursing Politics," and with respect to the rumours concerning the Registered Nurses' Society mentioned in the article in question, permit me to speak on behalf of the large majority of members who have *not* seceded. I learn that thirteen members and five probationers have withdrawn from the society. It may or may not be the case that the whole of that number, in consequence of depreciation of, and unfounded statements concerning, our society, which were industriously circulated, thought they would do well for themselves by joining our late secretary in her attempt to inaugurate a rival society. The great majority, however, of our members are too wise, and, I hope, too loyal, to desert their good co-operation, which has been so splendidly successful for two years, and to which they owe so much, simply because their late secretary decided to open at our very doors, with all the knowledge and experience she had gained while in our employment, an opposition society. While employed by the Registered Nurses' Society Miss Ella Jackson was always treated with the utmost kindness by the hon. treasurer, the hon. superintendent, and by the nurse members; as was acknowledged by herself just before leaving. It therefore caused much surprise that from time to time we heard reports, apparently systematically spread, that there were dissensions in the office, that the society was "going to smash" (I quote the exact words of the report as it reached me), and that all members who do not wish to lose their work would be wise to leave the R.N.S., and throw in their lot with the new "society." We have been obligingly told that we should all be accepted if we apply at once; having been a member of the Registered Nurses' Society is recommendation sufficient. I ask you, sir, in your impartial opinion, does this savour of common honesty? I read also in your paper a statement to the effect that at least half of the original committee of the R.N.S. form part of the new committee. I am very sorry to hear it. They withdrew from us some months ago, which, of course, they had every right to do if they wished, but I can scarcely believe that any member of the medical profession would lend his name in support of the proceedings which have recently taken place. I wish to say, in conclusion, that we members of the Registered Nurses' Society have worked hard and loyally for those doctors who have sought our services, and they have invariably expressed themselves satisfied with our work. There is good reason to believe that those gentlemen, with few, if any, exceptions, will continue their support to us so long as we continue to deserve it. In any case we —I speak the mind of the majority—intend to stand by the Registered Nurses' Society, and under no circumstances could we have part or lot in the new venture, founded, as we believe it to be, in disloyalty and ingratitude.

### HYGIENE FOR NURSES.

"A Reader" writes: In a very interesting article in The Hospital of April 11th, viz., "Hygiene and its Bearings: The House we Live in, in Relation to Health, Considered Generally," occurs the following sentence: "Nursing, worthy of the name, is absolutely impossible in houses of the poorer classes, and any kind of medical treatment is well-nigh valueless." In Liverpool, district nursing amongst the sick poor in their own homes is very encouraging on the whole; and as this city has been a sort of "pioneer" to the great scheme, we think it deserves to have a voice in the matter. Taking into consideration (in many cases) the spare amount of superficial and cubic space per individual, the imperfect means of ventilation, and very often defective sanitation, also the questionable morals of many patients, the results of nursing and medical treatment are satisfactory. A district nurse can enforce cleanliness and ventilation to a certain extent—at least until the patients' recovery, which is the point in question. The case may be one which has been denied a place in hospital, owing to its being a "hopeless" one; consequently the treatment is also hopeless. If the case be at all a "curable" one—for example, typhoid, pneumonia, uterine, or one requiring surgical treatment—the death of such or removal to hospital is considered rather a failure, after treatment at home has been tried. In a clean house, and where there are facilities for such, a successful little operation is not at all unusual. "Drink" is even a greater enemy than dirt in the "slums," and it is a well-known fact that a habitual drunkard has much less chance when attacked by disease, whereas their unclean surroundings do not affect them so much as one might think, their forefathers and their own generation having been accustomed to nothing else for ages. Altogether, nursing amongst the sick poor is most interesting, encouraging, and satisfactory in this quarter of the globe.

